# VP4 Mutation Boosts Replication of Recombinant Human/Simian Rotavirus in Cell Culture

**DOI:** 10.3390/v16040565

**Published:** 2024-04-05

**Authors:** Roman Valusenko-Mehrkens, Katja Schilling-Loeffler, Reimar Johne, Alexander Falkenhagen

**Affiliations:** Department of Biological Safety, German Federal Institute for Risk Assessment, 10589 Berlin, Germany; roman.valusenko-mehrkens@bfr.bund.de (R.V.-M.); katja.schilling-loeffler@bfr.bund.de (K.S.-L.); reimar.johne@bfr.bund.de (R.J.)

**Keywords:** rotavirus, Sub-Saharan Africa, reverse genetics system, triple-reassortant, point mutation, next-generation sequencing, replication kinetics, cell culture

## Abstract

Rotavirus A (RVA) is the leading cause of diarrhea requiring hospitalization in children and causes over 100,000 annual deaths in Sub-Saharan Africa. In order to generate next-generation vaccines against African RVA genotypes, a reverse genetics system based on a simian rotavirus strain was utilized here to exchange the antigenic capsid proteins VP4, VP7 and VP6 with those of African human rotavirus field strains. One VP4/VP7/VP6 (genotypes G9-P[6]-I2) triple-reassortant was successfully rescued, but it replicated poorly in the first cell culture passages. However, the viral titer was enhanced upon further passaging. Whole genome sequencing of the passaged virus revealed a single point mutation (A797G), resulting in an amino acid exchange (E263G) in VP4. After introducing this mutation into the VP4-encoding plasmid, a VP4 mono-reassortant as well as the VP4/VP7/VP6 triple-reassortant replicated to high titers already in the first cell culture passage. However, the introduction of the same mutation into the VP4 of other human RVA strains did not improve the rescue of those reassortants, indicating strain specificity. The results show that specific point mutations in VP4 can substantially improve the rescue and replication of recombinant RVA reassortants in cell culture, which may be useful for the development of novel vaccine strains.

## 1. Introduction

Rotaviruses are double-stranded RNA viruses which belong to the family *Sedoreoviridae* [1] and infect wild animals, livestock as well as humans [2]. Human Rotavirus A (RVA) can cause severe gastroenteritis in infants and young children and is the leading cause of diarrhea requiring hospitalization in children under five years of age in low- and middle-income countries [3]. In the absence of symptomatic treatment, an infection can become life-threatening due to dehydration [4]. Based on recent data, RVA causes over 100,000 annual deaths in Sub-Saharan Africa alone [5].

The RVA capsid contains three concentric protein layers. The inner layer is formed by VP2, the middle layer by VP6 and the outer layer by VP7 and the VP4 spike protein [6]. Each viral spike is composed of three VP4 molecules that are anchored to the virus particle by the interaction of the VP4 base with VP6 and VP7 [7,8]. Upon proteolytic cleavage of the spikes, VP4 is divided into the N-terminal receptor-binding fragment VP8* and the C-terminal membrane-penetrating fragment VP5*.

The rotavirus genome consists of 11 double-stranded RNA segments each encoding one or two viral proteins [1]. A classification system for rotaviruses based on the complete nucleotide sequences of all eleven genome segments has been established, enabling the precise description of reassortant strains, in which the genotypes of the genome segments VP7-VP4-VP6-VP1-VP2-VP3-NSP1-NSP2-NSP3-NSP4-NSP5 are represented by Gx-P[x]-Ix-Rx-Cx-Mx-Ax-Nx-Tx-Ex-Hx, respectively [9]. Upon co-infection of the same host cell with two different RVA strains, the discrete genome segments can be shuffled, resulting in a novel rotavirus strain consisting of a mixture of segments from both parental strains [10]. This reassortment event greatly contributes to a high diversity in circulating RVA strains. Especially the RVA genome segments encoding VP7 and VP4, which have a very high genetic variability. To date, 42 different VP7 genotypes and 58 different VP4 genotypes have been described [11]. 

VP4 and VP7 contain the major antigenic epitopes that elicit neutralizing antibody responses [12], but VP6 has also been shown to induce protective immunity [13,14,15]. There are two approved vaccines that have mainly been used in Africa (RotaTeq and Rotarix) [16]. RotaTeq is a pentavalent, live-attenuated vaccine consisting of five reassortants containing VP4 (P[8]) or VP7 (G1, G2, G3 or G4) from human RVA strains in a bovine RVA backbone [17]. In contrast, Rotarix is a live-attenuated vaccine derived from only one human G1P[8] RVA isolate [18]. Although the effectiveness of both vaccines ranges from 85-98% in America, Europe and parts of Asia, they show a reduced efficacy and effectiveness in low-income countries in Africa (50–64%) [19,20]. There are several possible reasons proposed for the reduced vaccine effectiveness, including malnutrition, host genetic factors such as histo-blood group antigens (HBGAs), differences in gut microbiota or co-infections with other pathogens [20]. However, the high diversity and difference of RVA strains circulating in Sub-Saharan Africa also have to be considered as possible reasons for a lower vaccine efficacy [21,22]. Additional recently licensed rotavirus vaccines are Rotavac (monovalent human G9P[11] RVA, developed in New Delhi, India), RotaSIIL (pentavalent bovine reassortants with human RVA G1–G4 and G9, developed in Pune, India), Rotavin-M1 (monovalent human G1P[8] RVA, developed in Hanoi, Vietnam) and Lanzhou (monovalent lamb G10P[12] RVA, developed in Lanzhou, China). The introduction of vaccines led to changes in circulating rotavirus strains and genotypes [23,24]. Before the rotavirus vaccine was introduced in South Africa, G1P[8] was the most detected genotype. However, the proportion of G1 strains decreased and the proportion of non-G1P[8] strains increased after the vaccine’s introduction [25], leading to a higher variety of circulating strains and an increase in uncommon genotype constellations. 

Human RVA strains are difficult to adapt to replication in cell culture, which limits the possibilities to investigate reassortment and generate vaccine strains. Recently, entirely plasmid-based reverse genetics systems for RVA have been developed [26,27]. These reverse genetics systems are based on transfecting cell lines that constitutively express T7 RNA polymerase with plasmids encoding each rotavirus genome segment under the control of the T7 RNA polymerase promoter followed by infection of a cell line that is susceptible to rotavirus infection. The utilization of these reverse genetics systems enabled the generation of several human RVA strains including G1P[8] KU, G4P[8] Odelia, G2P[4] HN126 or G1P[8] CDC-9 [27,28,29,30]. The developed reverse genetics systems were also used to investigate the reassortment of diverse animal rotavirus genome segments [30,31,32,33,34]. Additionally, several studies investigated the reassortment of the genome segment encoding VP4 from human RVA strains in the backbone of the simian RVA strain SA11, resulting in the generation of SA11 reassortants with VP4 from Odelia, CDC-9, HN126 or clinical isolates (P[4] or P[8]) [28,29,30,35]. However, according to the mentioned studies, reassortants with VP4 from human RVA strains tend to replicate poorly in cell culture. Recently, two studies investigated whether different combinations of human RVA genome segments encoding VP4, VP7 and VP6 in an SA11 backbone improved replication [29,35]. While one study showed that combining human RVA P[8] VP4 with homologous G1 VP7 or with homologous VP7 and VP6 did not improve the rescue of SA11 reassortants, another study reported that the interaction of human RVA P[4] VP4 with homologous G2 VP7 contributed to efficient virus infectivity.

Previously, we also investigated the generation of SA11 reassortants containing VP4 and/or VP7 from three African human RVA strains that have never been adapted to cell culture: GR10924 (G9P[6]), Moz60a (G12P[8]) and Moz308 (G2P[4]) [36]. The strains were chosen because they represented common genotypes circulating in Africa and complete sequence data were available. We were able to rescue SA11 mono-reassortants with VP7 from all three human RVA strains as well as one slowly replicating SA11 mono-reassortant with P[6] VP4 from GR10924. However, the rescue of SA11 double-reassortants containing VP4 and VP7 from human RVAs was not possible. Recently, we have shown that restoring the natural interactions between VP4, VP7 and VP6 from the human RVA strain Wa improved the rescue of SA11/Wa reassortants [37].

In the current study, we investigated whether restoring the natural interactions between VP4, VP7 and VP6 from the three African RVA strains would enable us to generate viable reassortants containing their main antigens. Although this approach was of limited success, one triple-reassortant was generated that changed its phenotype and started to replicate to higher titers after initial cell culture passages. The sequencing of its whole genome identified a unique point mutation in VP4, which could be shown to substantially improve virus rescue and replication. The results may contribute to the improved generation of specific recombinant rotaviruses and may be useful for the development of novel vaccine strains containing human RVA P[6] VP4.

## 2. Materials and Methods

### 2.1. Cell Lines and Viruses

All cell culture reagents and media were obtained from Pan-Biotech (Aidenbach, Germany) unless indicated otherwise. Dulbecco’s Modified Eagle’s Medium and Minimal Essential Medium were supplemented with 10% fetal bovine serum (FBS), 1× non-essential amino acids, 2 mM L-glutamine and 0.1 μg/mL gentamicin (hereafter referred to as DMEM and MEM, respectively). MA-104 cells were provided by the European Collection of Authenticated Cell Cultures (Salisbury, UK) and cultured in MEM. BSR-T7/5 cells were kindly provided by Dr. Karsten Tischer (Free University of Berlin, Berlin, Germany) and maintained in DMEM containing 1 mg/mL G418 (Biochrome, Berlin, Germany). All cells were incubated at 37 °C, 5% CO_2_ and 85% RH. The virus strain RVA/Simian-tc/ZAF/SA11-L2/1958/G3P[2], referred to as SA11, was generated by using the plasmid-based reverse genetics system as described below.

### 2.2. Plasmids

The plasmids encoding the eleven SA11 genome segments, as well as the three helper plasmids pCAG-D1R, pCAG-D12L and pCAG-FAST-p10 encoding the vaccinia virus capping enzyme subunits D1R and D12L as well as the small fusion protein FAST were kindly provided by Takeshi Kobayashi [26] and obtained from Addgene (Watertown, MA, USA). The generation of the plasmids encoding VP4 and VP7 from the African human RVA strains RVA/Human-wt/ZAF/GR10924/1999/G9P[6], RVA/Human-wt/MOZ/0060a/2012/G12P[8] and RVA/Human-wt/MOZ/0308/2012/G2P[4] (referred to as GR10924, Moz60a and Moz308, respectively) has been described previously [36]. Expression cassettes containing a T7 RNA polymerase promoter, VP6 from human RVA strain GR10924, Moz60a or Moz308 (GenBank acc.-no. FJ183358.1, MG926762.1 or MG926729.1, respectively) [38,39], the hepatitis delta virus ribozyme and a T7 terminator were synthesized by Integrated DNA Technologies (IDT, Coralville, IA, USA) as dsDNA fragments. The promoter, hepatitis delta virus ribozyme and terminator sequence were identical to a plasmid described previously (Genbank: KT239165) [40]. The expression cassettes were cloned into pUC-IDT-Amp (IDT) using standard cloning techniques and sequence verified by Sanger sequencing (Eurofins Genomics GmbH, Ebersberg, Germany). Sequencing primers are available upon request. The VP4-encoding plasmid of GR10924 containing the mutation A797G was generated as described below. All plasmids were purified using the QIAfilter Plasmid Midi Kit (Qiagen GmbH, Hilden, Germany).

### 2.3. Plasmid-Based Reverse Genetics System

BSR-T7/5 cells were seeded in a 6-well plate (3.5 × 10^5^ cells per well) and incubated for 24 h. At 90% confluency, the cells were co-transfected with the eleven plasmids encoding the individual rotavirus genome segments and the three helper plasmids (2250 ng for the NSP2 and NSP5 encoding plasmids; 15 ng for the FAST-encoding plasmid and 750 ng for the remaining plasmids) using 30 µL of TransIT-LT1 transfection reagent (Mirus Bio, Madison, WI, USA). The transfected cells were incubated for 24 h before they were washed once with DMEM without FBS. Next, DMEM without FBS containing 0.5 µg/mL trypsin (Pan-Biotech) was added. After an additional 48 h of incubation, the transfected BSR-T7/5 cells were co-cultured with MA-104 cells (1 × 10^5^ cells per well) in the presence of trypsin (2 μg/mL final concentration). After three days, the co-cultured cells including the culture media were frozen at −20 °C and thawed at room temperature. After low-speed centrifugation, clarified supernatants, referred to as freeze/thaw supernatants throughout the manuscript, were collected and used to infect MA-104 cells as described below.

### 2.4. Passaging of Reassortants

The reassortant viruses were essentially passaged as described previously [41,42]. In brief, for the first passage, trypsin (Pan-Biotech) was added to the entire (~2 mL) clarified freeze/thaw supernatants (final concentration: 20 µg/mL) from co-cultures of transfected BSR-T7/5 and MA-104 cells. These infection mixtures were incubated for one hour at 37 °C. Confluent MA-104 cells grown in a 6-well plate were washed twice with PBS and the infection mixtures were added. After an additional hour of incubation at 37 °C, the mixtures were removed from the cells, fresh MEM without FBS containing trypsin (final concentration: 2 µg/mL) were added, and the cells were incubated for seven days. For later passages, clarified freeze/thaw supernatants (~2 mL) were collected as described in Section 2.3 and 150 µL samples were taken for RNA analyses. The remaining clarified freeze/thaw supernatants were used to infect fresh MA-104 cells as described above.

### 2.5. RNA Extraction, qRT-PCR, RT-PCR and Sanger Sequencing

Viral RNA was extracted from freeze/thaw supernatants with the NUCLISENS easyMAG system (bioMérieux, Marcy-l’Étoile, France) and digested with RNase-free DNase (Roche, Basel, Switzerland) according to the manufacturer’s instructions before analyses by qRT-PCR or RT-PCR. The qRT-PCR was performed as described previously [43]. To determine the number of genome copy equivalents (GCEs)/mL culture supernatant, qRT-PCR analyses were performed with RNA isolated from culture supernatants and a pT7-NSP3SA11 plasmid standard with a known copy number. A quantification example is shown in Supplementary Figure S1. RT-PCR analyses were used to determine the presence of the expected virus genome segments and performed using the OneStep RT-PCR Kit (Qiagen, Hilden, Germany) with primers as listed in Supplementary Table S1, according to the manufacturer’s instructions. After RT-PCR, 1 µL of 5× DNA Loading Buffer, Blue (meridian Bioscience, Cincinnati, OH, USA) was added to the RNA, loaded onto a 2% agarose gel and separated at 100 V for 1 h. The gel was stained using ethidium bromide (Carl Roth, Karlsruhe, Germany) and visualized under UV light. For Sanger sequencing, PCR amplicons were cleaned up using the Monarch DNA Gel Extraction Kit (New England BioLabs, Ipswich, UK) and sent to Eurofins Genomics GmbH.

### 2.6. Whole Genome Sequencing and Sequence Analysis

Nucleic acid extracts (see Section 2.5) were used for preparing libraries with the KAPA RNA HyperPrep Kit (Roche Diagnostic, Mannheim, Germany) and the KAPA Unique Dual-Indexed Adapter Kit for Illumina^®^ platforms (Roche Diagnostic) as previously described [44]. Resulting libraries were sequenced along with 119 libraries with 2 × 150 cycles using the NextSeq 500/550 Mid Output Kit v2.5 (Illumina, San Diego, CA, USA) on the NextSeq 500 Sequencer (Illumina). All of the sequence analysis was performed in Geneious Prime^®^ 2023.2.1 (Biomatters Ltd., Auckland, New Zealand). Raw reads were trimmed using the BBDuk plugin. Segment sequences were assembled with the map to reference function, where eleven selected segment sequences were used as references (accession numbers: LC178570-LC178574, LC178564-LC178566, FJ183356, FJ183358 and FJ183360).

### 2.7. Site-Directed Mutagenesis

To introduce the point mutation A797G into the plasmids encoding VP4 from human RVA strains GR10924, Moz60a and Moz308, the Phusion Site-Directed Mutagenesis Kit (Thermo Fisher Scientific, Waltham, MA, USA) was used in accordance with the manufacturer’s instructions. In brief, 5 ng of the targeted plasmid was amplified by PCR with two 5′-phosphorylated primers. The primers were designed to anneal back-to-back to the plasmid and the desired mutation was introduced into the forward primer. Primer sequences are shown in Supplementary Table S1. The PCR conditions were: Initial denaturation at 98 °C for 30 s (step 1); denaturation at 98 °C for 10 s (step 2); annealing at 65 °C for 20 s (step 3); extension at 72 °C for 150 s (step 4); final extension at 72 °C for 5 min (step 5). Step 2 to step 4 were repeated 25 times. After the digestion of parental methylated and hemimethylated DNA with FastDigest DpnI, the PCR product containing the mutation was circularized by ligation with T4 DNA Ligase. Finally, chemically competent One Shot^®^ TOP10 E. coli (Thermo Fisher Scientific) was transformed as instructed by the manufacturer. For verification of the introduced mutation, the corresponding region of the plasmid was amplified by PCR using primers listed in Supplementary Table S1 and the PCR products were subjected to Sanger sequencing (Eurofins Genomics GmbH).

### 2.8. Replication Kinetics

Confluent MA-104 cells grown in 6-well plates were infected with cell culture supernatants containing viruses at 2 × 10^4^ GCEs as described above. At the indicated time points, 500 µL samples were taken and the same volume of fresh media containing 2 µg/mL trypsin was added. Once all the samples were collected, viral RNA was extracted, digested with RNase-free DNase and analyzed by qRT-PCR as described above.

### 2.9. Sequence Analyses and Protein Structure Visualization

Sequences were constructed and analyzed with the SeqBuilder Pro software (Version 17.0.2; DNASTAR Inc., Madison, WI, USA). Alignments were performed using the MUSCLE method as implemented in MegAlign Pro (DNASTAR Inc.). Amino acid sequences were deduced using the SeqBuilder Pro software and the NCBI non-redundant protein sequences data base was screened using BLASTp search (https://blast.ncbi.nlm.nih.gov/Blast.cgi (accessed on 14 March 2024)). Protein structures were visualized and analyzed using Protean 3D (DNASTAR Inc.) or UCSF Chimera (University of California, San Francisco, CA, USA) [45] on the basis of the published atomic model of an infectious rotavirus particle (PDB 4v7q) [7].

### 2.10. Statistics

The data are presented as mean ± standard deviation. To determine statistical significance, a two-tailed unpaired *t*-test was used. Results with a *p*-value below 0.05, 0.01 or 0.001 were considered statistically significant and marked with one, two or three asterisks, respectively.

## 3. Results

### 3.1. Generation of Triple-Reassortants Carrying VP4, VP7 and VP6 from African Human Rotavirus A Strains

**Figure 1:**
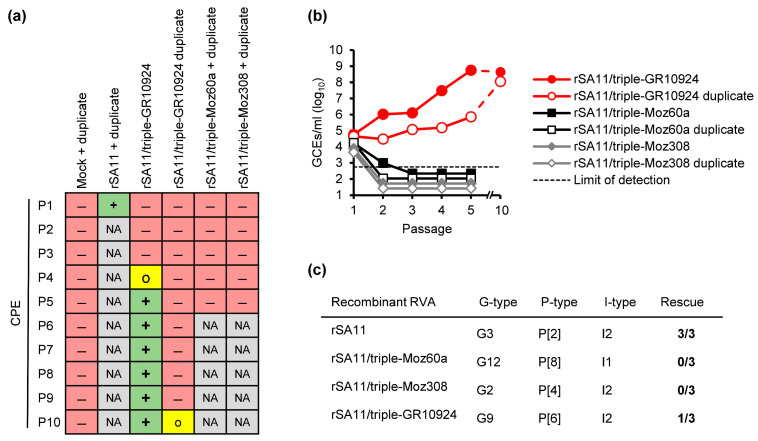
Generation of SA11 triple-reassortants containing VP4, VP7 and VP6 from three African human RVA strains. (**a**) Overview of the developed cytopathic effect (CPE) upon passaging in MA-104 cells. (**b**) Analyses of the freeze–thaw supernatants by qRT-PCR after the indicated passages in MA-104 cells. (**c**) Overview of the VP7 (G-type), VP4 (P-type) and VP6 (I-type) genotypes and the number of successful rescue experiments. The first rescue experiment was performed in duplicates but counted as one experiment. Mock = Mock-infected cells; rSA11 = Recombinant SA11; rSA11/triple-GR10924, rSA11/triple-Moz60a and rSA11/triple-Moz308 = Recombinant rotaviruses carrying segment 4 (VP4), segment 9 (VP7) and segment 6 (VP6) from the indicated human RVA strain in the backbone of SA11; P1–10 = Passages 1–10; red minus = No CPE; yellow O = Mild CPE; green plus = Strong CPE; NA = Not analyzed; GCEs = Genome copy equivalents.

We aimed to perform plasmid-based reverse genetics for RVA using simian RVA strain SA11 as a backbone and replacing the genome segments encoding for VP4 (segment 4), VP7 (segment 9) and VP6 (segment 6) with the corresponding segments from African RVA strain Moz60a (rSA11/triple-Moz60a), Moz308 (rSA11/triple-Moz308) or GR10924 (rSA11/triple-GR10924). Rescue of recombinant simian RVA strain SA11 (rSA11) served as a positive control. BSR-T7/5 cells were transfected with the respective plasmids in duplicates and then co-cultured with MA-104 cells. Freeze/thaw supernatants from co-cultured cells were passaged on MA-104 cells and the inoculated cells were monitored for signs of an RVA-typical cytopathic effect (CPE). Figure 1a depicts an overview of the development of CPEs upon passaging in MA-104 cells. 

For rSA11 and the rSA11 duplicate, a clear cytopathic effect (CPE) was evident after the first passage, indicating that rescue was successful. However, no CPE was observed for any reassortant after passage 1. Cells infected with rSA11 were discarded to reduce the risk of cross-contamination, while all reassortants were passaged until passage 5. After the fifth passage, viral RNA was extracted from freeze/thaw supernatants of passage 1 to passage 5 and analyzed by qRT-PCR. No CPE was evident for rSA11/triple-Moz60a, rSA11/triple-Moz308 or their duplicates. In contrast, a CPE developed for rSA11/triple-GR10924 in the fourth passage and was clearly observable by passage 5, but no CPE was observed for the rSA11/triple-GR10924 duplicate by passage 5. Analyses by qRT-PCR showed that RVA RNA could be detected for rSA11/triple-GR10924 and the rSA11/triple-GR10924 duplicate after each passage, but the RNA titer of the reassortant that caused a CPE was higher after passages 2–5 (Figure 1b). For rSA11/triple-Moz60a, rSA11/triple-Moz308 and their duplicates, RVA RNA declined after the first passage and was not detectable anymore by passage 3 (Figure 1b), suggesting that the rescue of these triple-reassortants failed. Both rSA11/triple-GR10924 and the rSA11/triple-GR10924 duplicate were further passaged on MA-104 cells until passage 10, by which time the duplicate started to develop a CPE and the RNA titers increased (Figure 1a,b), indicating the successful rescue of rSA11/triple-GR10924 and the duplicate. Rescue experiments were repeated two additional times for each reassortant, but not in duplicates. While similar results were obtained for rSA11/triple-Moz60a and rSA11/triple-Moz308, we were unable to re-rescue the rSA11/triple-GR10924 reassortant (Supplementary Figure S2), suggesting that the rescue of this reassortant is possible but inefficient using the reverse genetics system. Those experiments were stopped after passage 3 as the first rescue experiment showed that detection of RVA RNA corresponded with successful rescue at this passage number.

### 3.2. Next-Generation Sequencing Revealed Point Mutations in rSA11/triple-GR10924 and the Duplicate

**Figure 2:**
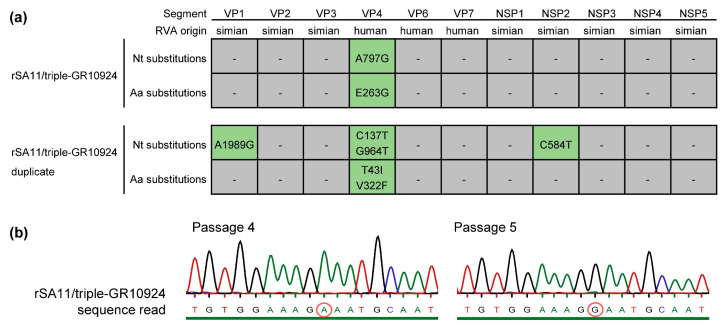
Sequence analyses of rSA11/triple-GR10924 and the duplicate. (**a**) Nucleotide (Nt) and amino acid (Aa) substitutions in the open reading frames of the eleven rotavirus genome segments identified by next-generation sequencing after passage 10. (**b**) Sanger sequencing analyses of the VP4-encoding genome segment from the rSA11/triple-GR10924 reassortant that replicated to a higher titer in early passages. Respective sequencing chromatograms of passage 4 and 5 are shown. The red circle marks nucleotide position 797 in the VP4-encoding genome segment from human RVA strain GR10924. The green line below the chromatograms indicates that the probability for a wrong base call was equal to or less than 1 in 1000. rSA11/triple-GR10924 = Recombinant rotavirus carrying segment 4 (VP4), segment 9 (VP7) and segment 6 (VP6) from human RVA strain GR10924 in the backbone of SA11.

After ten passages in MA-104 cells, the rSA11/triple-GR10924 and the rSA11/triple-GR10924 duplicate were analyzed by whole genome next-generation sequencing. For rSA11/triple-GR10924 and the duplicate, 2,268,340 and 2,986,102 reads were obtained, respectively. An overview of the average coverage, coverage range and the percentage of the open reading frame (ORF) sequenced is shown in Supplementary Table S2. The complete ORFs of each genome segment were covered, with the exception of genome segment 4 (VP4) from the rSA11/triple-GR10924 duplicate, where the first two nucleotides of the ORF could not be sequenced. For the rSA11/triple-GR10924 reassortant that replicated to a higher titer in early passages, only a single point mutation located in the ORF of genome segment 4 (VP4) was identified. The adenine at position 797 was substituted by a guanine, which led to an amino acid substitution of glutamic acid to glycine at position 263. For the duplicate, four mutations were located in ORFs: A1981G in genome segment 1 (VP1); C137T and G964T in genome segment 4 (VP4); C584T in genome segment 8 (NSP2). However, only the mutations detected in the VP4 ORF were non-synonymous, resulting in amino acid substitutions T43I and V322F. An overview of the identified mutations is depicted in Figure 2a.

Additionally, partial untranslated region (UTR) sequences were obtained. The Supplementary Tables S3 and S4 show the identified UTR sequences for the genome segments encoding structural and non-structural proteins, respectively. No nucleotide substitutions were detected in the UTRs of rSA11/triple-GR10924, but one nucleotide substitution located in the 3′UTR of genome segment 10 encoding NSP4 (T705C) was identified for the duplicate. 

The point mutation in the rSA11/triple-GR10924 reassortant that replicated to a higher titer in early passages was further characterized. First, the presence of the mutation in passage 10 virus was confirmed by Sanger sequencing. Analysis of passages 1–5 by Sanger sequencing showed that the virus with the mutation A797G (E263G) in VP4 became predominant in the fifth passage. Figure 2b shows VP4 sequencing chromatograms of the corresponding region from passage 4 and passage 5 virus. Sequencing results from passages 1–3 are shown in Supplementary Figure S3. 

### 3.3. Introduction of Mutation A797G into the VP4-Encoding Plasmid of GR10924 Improves Rescue of Reassortants

**Figure 3:**
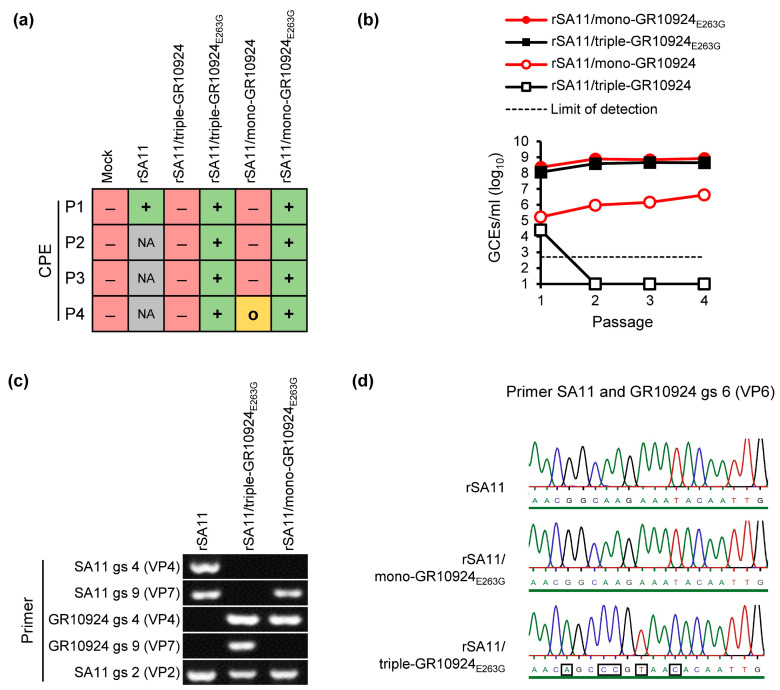
Rescue of SA11 mono- and triple-reassortants containing VP4 from human RVA strain GR10924 with and without the mutation A797G in the VP4-encoding genome segment. (**a**) Cytopathic effect (CPE) upon passage in MA-104 cells. (**b**) Determined number of genome copy equivalents (GCEs)/mL in freeze–thaw supernatant after each passage. (**c**) Detection of VP4- and VP7-encoding genome segments from rSA11 and rescued reassortants via RT-PCR using strain- and genome segment-specific primer pairs followed by agarose gel electrophoresis analysis. (**d**) Detection of VP6-encoding genome segments from rSA11 and rescued reassortants via RT-PCR using VP6-specific primer pairs followed by Sanger sequencing. The black squares mark nucleotide differences between the VP6-encoding genome segment from SA11 and GR10924. The green line below the chromatograms indicates that the probability for a wrong base call was equal to or less than 1 in 1000. rSA11 = Recombinant SA11; rSA11/triple-GR10924 = Recombinant rotavirus carrying segment 4 (VP4), segment 9 (VP7) and segment 6 (VP6) from human RVA strain GR10924 in the backbone of SA11; rSA11/triple-GR10924_E263G_ = rSA11/triple-GR10924 with VP4-E263G; rSA11/mono-GR10924 = Recombinant rotavirus carrying segment 4 (VP4) from human RVA strain GR10924 in the backbone of SA11; rSA11/mono-GR10924_E263G_ = rSA11/mono-GR10924 with VP4-E263G; gs = genome segment; P1–4 = Passages 1–4; red minus = No CPE; yellow O = Mild CPE; green plus = Strong CPE; NA = Not analyzed; GCEs = Genome copy equivalents.

To determine whether VP4-E263G affects the rescue of reassortants, the A797G mutation was introduced into the VP4-encoding plasmid of GR10924 by site-directed mutagenesis. Rescue experiments to generate rSA11/triple-GR10924 without and with VP4-E263G (rSA11/triple-GR10924_E263G_) were performed as described above. An overview of the appearance of CPEs during each passage is shown in Figure 3a. After the first passage, a CPE appeared in rSA11/triple-GR10924_E263G_. In contrast, rSA11/triple-GR10924 without the mutation did not develop a CPE by the end of passage 4. Analyses of freeze/thaw supernatants collected at the end of every passage by qRT-PCR revealed that rSA11/triple-GR10924_E263G_ already replicated to a high titer in the first passage, while rSA11/triple-GR10924 without the mutation could not be rescued again (Figure 3b).

We have previously been able to generate a recombinant rotavirus containing genome segment 4 (VP4) from human RVA strain GR10924 in the backbone of SA11 (rSA11/mono-GR10924) using a similar reverse genetics approach, but poor replication was observed in MA-104 cells [36]. To analyze whether VP4-E263G also improved the rescue of this mono-reassortant, the generation of rSA11/mono-GR10924 without and with VP4-E263G (rSA11/mono-GR10924_E263G_) was attempted. While a CPE was only evident in passage 4 for rSA11/mono-GR10924 without the mutation, a CPE could already be detected in passage 1 for rSA11/mono-GR10924_E263G_ (Figure 3a). The rSA11/mono-GR10924_E263G_ reassortant also reached higher RNA titers than the reassortant without the mutation after passages 1–4 (Figure 3b). All rescue experiments were repeated with similar results for the rSA11/triple-GR10924, rSA11/triple-GR10924_E263G_ and rSA11/mono-GR10924_E263G_, but the second rescue attempt of the rSA11/mono-GR10924 reassortant without the mutation was not successful (Supplementary Figure S4), suggesting that the rescue of this reassortant was also not efficient using the reverse genetics system employed here. 

In order to confirm the identity of the rescued reassortants, RVA RNA from the generated reassortants was analyzed by RT-PCR using specific primer pairs for the genome segments encoding VP4 and VP7 from human RVA GR10924 as well as for the segments encoding VP4, VP7 and VP2 from simian RVA SA11. Analyses of the resulting PCR products by agarose gel electrophoresis confirmed that the expected genome fragments were present for each reassortant (Figure 3c). To confirm that the expected VP6-encoding genome segment was present in the rescued viruses, a primer pair that could bind to the VP6-encoding segment from SA11 and GR10924 was used in RT-PCR analyses and the PCR products were analyzed by Sanger sequencing (Figure 3d).

Having shown that the amino acid substitution E263G improved the rescue of reassortants containing VP4 from GR10924, we were interested in examining whether the mutation also improved the rescue of recombinant rotaviruses with VP4-encoding segments from the other African human RVA strains in the backbone of SA11. The mutation was introduced into the VP4-encoding plasmids of Moz60a and Moz308. However, rescue of Moz60a or Moz308 triple-reassortants with VP4-E263G (rSA11/triple-Moz60a_E263G_ or rSA11/triple-Moz308_E263G_, respectively) as well as mono-reassortants with VP4-E263G (rSA11/mono-Moz60a_E263G_ or rSA11/mono-Moz308_E263G_, respectively) was not successful, as indicated by the absence of a CPE (Supplementary Figure S5a) and RVA RNA (Supplementary Figure S5b) after four passages in MA-104 cells.

### 3.4. VP4-E263G Improves Replication of Reassortants

**Figure 4:**
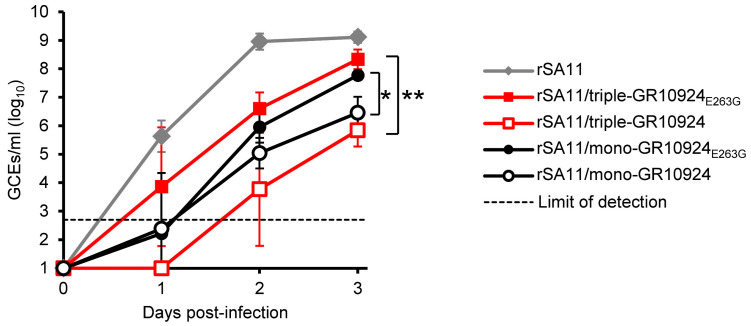
Replication kinetics in MA-104 cells. Cells were infected with 2 × 10^4^ genome copy equivalents (GCEs) corresponding to 0.04 GCEs/cell and the number of GCEs in culture supernatants was determined by qRT-PCR at the indicated time points post-infection. Data are means ± standard deviation from three independent experiments. rSA11 = Recombinant SA11; rSA11/triple-GR10924 = Recombinant rotavirus carrying segment 4 (VP4), segment 9 (VP7) and segment 6 (VP6) from human RVA strain GR10924 in the backbone of SA11; rSA11/triple-GR10924_E263G_ = rSA11/triple-GR10924 with VP4-E263G; rSA11/mono-GR10924 = Recombinant rotavirus carrying segment 4 (VP4) from human RVA strain GR10924 in the backbone of SA11; rSA11/mono-GR10924_E263G_ = rSA11/mono-GR10924 with VP4-E263G; ** *p* < 0.01 for rSA11/triple-GR10924_E263G_ versus rSA11/triple-GR10924 on day 3. * *p* < 0.05 for rSA11/mono-GR10924_E263G_ versus rSA11/mono-GR10924 on day 3.

In order to determine the growth kinetics of the generated reassortants and to investigate whether VP4-E263G improves replication, MA-104 cells were infected with rSA11 and reassortants containing VP4 with and without the mutation. As we were unable to rescue rSA11/triple-GR10924 without any mutation in the VP4-encoding genome segment, the rSA11/triple-GR10924 duplicate that contained two other amino acid substitutions in VP4 (see Section 3.2) was used in this experiment. MA-104 cells were infected with rSA11 and the respective reassortants using an equal number of GCEs, and cell culture supernatants were collected at indicated time points post-infection, and the number of GCEs/mL was determined by qRT-PCR (Figure 4). While higher mean titers were observed for the rSA11/mono-GR10924_E263G_ in comparison to the rSA11/triple-GR10924 without the mutation throughout the experiment, the individual titers varied considerably on day 1 and day 2 post-infection. Titers became more consistent by day 3 post-infection. At that time point, the titer of the rSA11/triple-GR10924_E263G_ was 2.5 log_10_ higher than the titer of rSA11/triple-GR10924 without the mutation (*p* < 0.01). Similarly, the titer of rSA11/mono-GR10924_E263G_ was 1.3 log_10_ higher than the titer of rSA11/mono-GR10924 without the mutation (*p* < 0.05) on day 3 post-infection.

### 3.5. Searching for the Presence of E263G, T43I and V322F Exchanges in Reported RVA Field Strain Sequences

The NCBI non-redundant protein sequences data base was screened by BLASTp search using the deduced complete amino acid sequence of the wildtype GR10924 VP4. It was found that no sequence containing the E263G exchange was present in the 100 most closely related hits, indicating that the mutation is not common in field strains. To search more specifically for the mutation, a BLASTp search was performed with a short amino acid sequence (residues 252–272 of GR10924 mVP4) including the E263G exchange. This search resulted in 3/100 hits which contained the E263G exchange. This included two human P[6] strains from Mali (AB938246) [46] and India (EU753965) [47], as well as one human P[8] strain from China [48]. In all cases, the amino acid exchange resulted from an A797G mutation in the VP4 gene. A BLASTp search was also performed with short amino acid residues 33–53 and 312–332 of GR10924 VP4 including the T43I and V322F exchanges, respectively. This search only identified one human P[6] strain with T43I from South Korea (KF650088) [49] and no hit for V322F. 

## 4. Discussion

Cell culture isolation of human RVA strains is difficult and often not successful, leading to a lack of cell culture isolates for many important human RVA genotypes and antigenetic variants. The utilization of the recently established, plasmid-based reverse genetics system facilitates the generation of recombinant rotaviruses, which can also contain antigens of several human RVA genotypes. Using that system, we have previously generated diverse reassortants with VP4, VP7 and VP6 in various combinations from human and non-human rotavirus strains [31,32,33,34,36,37]. However, the generation of reassortants containing VP4 of human wildtype RVA was often not successful or resulted in only slowly replicating viruses. Here, we adapted our latest insights to rotavirus strains that were identified in Sub-Saharan Africa. By restoring the natural human RVA VP4, VP7 and VP6 interaction, we hoped to improve the rescue of these strains. However, we were only able to rescue one of those reassortants, rSA11/triple-GR10924. The rescue of rSA11/triple-GR10924 was inconsistent using our reverse genetics approach and replication to higher titers was linked to the development of mutations in VP4, of which the A797G mutation identified by NGS occurred at an early passage (P5) and was confirmed by reverse genetics. The results indicate that single-point mutations in the VP4 gene of human wildtype RVA can substantially improve cell culture replication. In addition, the availability of well-replicating reassortants with human RVA P[6] VP4 may be useful for basic and applied research.

We have previously rescued an SA11 mono-reassortant carrying VP4 from human RVA strain GR10924, but the rescue of SA11 double-reassortants containing both VP4 and VP7 from GR10924 was unsuccessful [36]. We have also shown that combining the genome segments encoding VP4, VP7 and VP6 from the cell culture-adapted human RVA strain Wa in the backbone of SA11 improved the rescue of reassortants [37]. Here, we generated SA11 containing VP4 and VP7 from GR10924 by including VP6 from GR10924. However, the rescue of SA11 triple-reassortants containing VP4, VP7 and VP6 from two other human RVA strains of African origin was not successful. Kanai et al. also tested the generation of SA11 reassortants carrying combinations of VP4, VP7 and VP6 from clinical isolate U14 in the backbone of SA11 [35]. While a poorly replicating mono-reassortant with VP4 from U14 was generated, rescue attempts of an SA11 double-reassortant with VP4 and VP7 from U14 or SA11 triple-reassortant with VP4, VP7 and VP6 from U14 were unsuccessful. In contrast, Hamajima et al. showed that the interplay of VP4 and VP7 from human RVA strain HN126 was important for the generation of SA11 reassortants [29]. Taken together, these results confirm that the generation of reassortants highly depends on the interaction of the capsid proteins, but that their interplay is complex and simply restoring the natural VP4, VP7 and VP6 interaction is not sufficient in all cases. Other factors could be the varying ability of VP4 from different wildtype human RVA strains to mediate entry into target cells, as shown by exchanging the receptor-binding fragment of VP4 with that of a cell culture-adapted strain [31]. In addition, interaction of VP6 with VP2 in the mature virus particles and VP6 interaction with NSP4 during virus assembly [50] may interfere with the generation of viable reassortants. 

The two rescued rSA11/triple-GR10924 reassortants developed non-synonymous mutations in VP4 within ten passages in MA-104 cells, suggesting that there was selective pressure on VP4. Indeed, most amino acid residue substitutions seem to be detected in VP4 upon long-term passaging of human RVA strains in cell culture [51]. However, the occurrence of a single amino acid sequence exchange in a very early passage number that coincided with a steep increase in titer was intriguing. In comparison, when human RVA strain CDC-9 was grown in MA-104 cells to passage 11 or 12, no nucleotide sequence changes from the original virus in stool were detected [52]. Interestingly, the single-point mutation that caused a titer increase was not detectable by Sanger sequencing at the end of passage 4, but it was predominant at the end of passage 5. As we were using nearly the entire clarified freeze/thaw culture supernatants for passaging, plaque-purifying the virus from early passages could not be performed, but should be considered for future rescue experiments to identify minor virus sub-populations. One possible explanation for the sudden dominance of the reassortant with the mutation in VP4 after passage 5 may be that only a small proportion of the virus without the mutation was able to infect MA-104 cells going from one passage to the next, while the virus with the mutation was much more efficient at infecting MA-104 cells, resulting in the selection of the virus with the mutation. By introducing the mutation identified here into the VP4-encoding plasmid of GR10294 and performing rescue experiments as well as replication kinetics analyses, we could confirm that this mutation substantially improved rescue and replication in MA-104 cells. This improvement was only found for VP4 from GR10924, but it did not improve virus rescue when introduced into VP4 of strains Moz60a and Moz308, indicating strain specificity. 

The mutation at VP4 position 263 was not present in other human RVA strains that have been continuously passaged in cell culture, e.g., CDC-9, Wa, M or the Rotarix pre-curser vaccine strain 89–12 [52,53,54]. However, mutations in close proximity (K262R, N267D and R268T) were identified in a study comparing the cell culture adaptation of three human strains in two different cell lines [51]. In addition, we could identify three human wildtype RVA strains which contained the E263G exchange [46,47,48] by screening of the NCBI sequence database. In each case, E263G was caused by an A797G mutation also found in passaged rSA11/triple-GR10924. This indicates that this mutation may be rare in field strains, but as it can be found in some of them it seems to also support replication in humans. In contrast, we could only identify one field strain containing the T43I exchange [49] and no strain that contained V322F, which indicates that these mutations are very rare in field strains.

**Figure 5:**
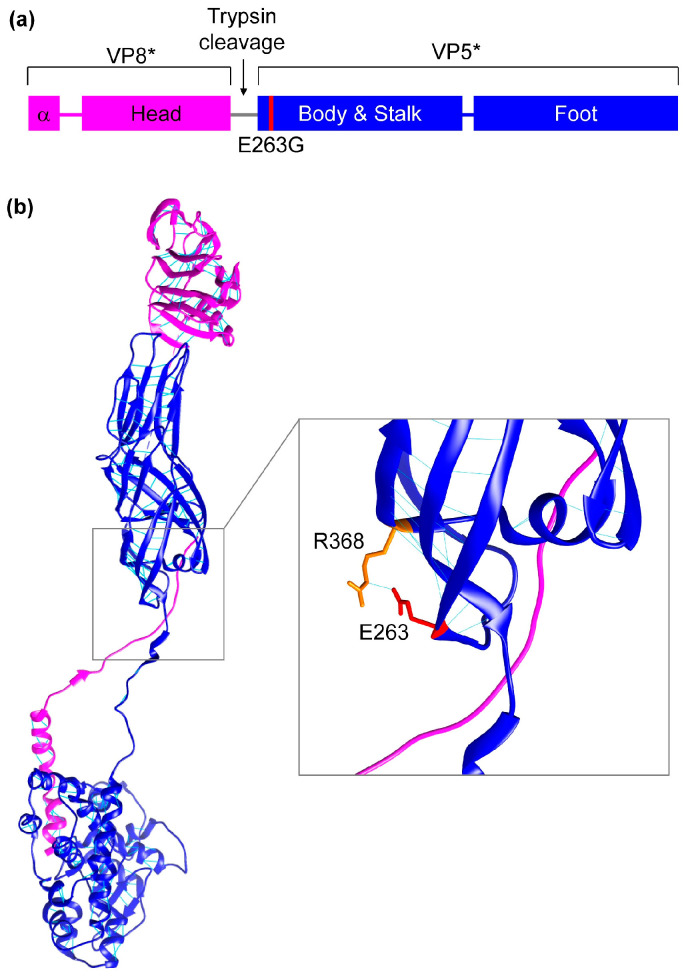
Location of E263 in VP4. (**a**) Schematic of VP4. VP4 is cleaved by trypsin into VP8* and VP5*. The location of the trypsin cleavage site is indicated. VP8* is composed of an α-helix at the N-terminus followed by the head region. The N-terminal helix interacts with the foot region of VP5* and the head region contains the putative receptor-binding site. VP5* contains the body and stalk region at the N-terminus and the foot region at the C-terminus. The location of E263G is shown in red. (**b**) Three-dimensional structure of VP4 on the basis of the atomic model of an infectious rhesus rotavirus (RRV) particle (PDB 4v7q, chain BX). VP8* is colored in magenta and VP5* in blue. Predicted hydrogen bonds are in cyan. The location of E263 in VP4 from GR10924 corresponding to E264 in VP4 from RRV is highlighted in red. R368 in VP4 from GR10924 corresponding to R369 in VP4 from RRV is shown in orange.

It is unclear how the amino acid substitution VP4-E263G improved replication. Glutamic acid is a comparatively large, bulky and rigid amino acid that is negatively charged. Meanwhile, glycine is the smallest amino acid, which is very flexible and does not have a charge. There could be multiple explanations for the observed enhancement in replication. VP4 is proteolytically cleaved by trypsin into VP8* and VP5*. The amino acid residue E263 is downstream of the trypsin cleavage site (residues 231–248 of VP4 from human RVA strain GR10924) and located at the N-terminal region of VP5* (Figure 5a). As E263G is distant from the cleavage site, an effect on proteolytic cleavage seems unlikely, but trypsin cleavage analysis of VP4-E263G would be required to exclude this possibility with certainty.

VP5* plays a role in the perforation of the cellular membrane, but Dowling et al. have shown that VP5* deletion mutants containing residues 265 to 474 or 265 to 404 of VP4 still retained cell permeabilization capabilities [55], suggesting that VP4-E263G is at least not directly affecting cell permeabilization. However, membrane penetration requires the VP4 spike to undergo conformational changes from an upright to a folded-back structure following attachment mediated by VP8* [6,56]. Jenni et al. have reported that mutations in VP5* had a stabilizing effect on the upright conformation of VP4, which resulted in increased infectivity of human RVA CDC-9 [57]. E263G could also lead to changes in the VP4 conformation. Protein structure analysis of the single VP4 molecule (Figure 5b) predicted that a hydrogen bond is formed between glutamic acid at position 264 and arginine at position 369 of VP4 from RRV, which corresponds to glutamic acid at position 263 and arginine at position 368 of VP4 from GR10924, respectively. 

Additionally, the rotavirus spike is formed by three VP4 subunits (VP4A, VP4B and VP4C). In VP4A and VP4B, the amino acid residue corresponding to E263 from GR10924 is distant from VP7 or VP6. However, E263 in VP4A is in contact with VP4B and vice versa (Figure S6a). Interestingly, the mutation T42I identified in the rSA11/triple-GR10924 duplicate is close to the same VP4A/VP4B contact site (Figure S6a), which could alter the VP4 subunit interaction with each other. The V322F mutation identified in the duplicate is distant from other VP4 subunits, VP7 or VP6 in VP4A and VP4B (Figure S6a). In VP4C, the N-terminal tip of VP5* lays in a gap formed by two VP7 trimers. In this conformation, it has previously been reported that residue 267 of RRV VP4 (corresponding to residue 266 of GR10924 VP4) is in contact with the RRV VP7 loop containing residue 200 [7]. The residue T42 was not resolved in the VP4C structure, but V322 was in proximity to the N-terminal region of VP4B (Figure S6b). It is of note that, aside from affecting the virion structure and entry, mutations could also impact other VP4 functions. For example, it has recently been shown that VP4 interacts with actin and facilitates the viroplasm assembly process [58,59].

Reverse-engineered rotaviruses could serve as next-generation rotavirus vaccine strains and recent studies have already explored the generation of recombinant rotaviruses that express foreign immunogens to use as multivalent vaccine vectors [60,61,62,63]. The rSA11/triple-GR10924_E263G_ reassortant generated in our study replicated to high titers in cell culture and could have potential as a vaccine candidate as there are currently no approved vaccines that contain human P[6] VP4, although the rotavirus vaccine candidate RV3-BB (monovalent human G3P[6] RVA, Parkville, Australia) is being tested in in Blantyre, Malawi [64,65]. VP4 contains multiple antigenic epitopes that induce neutralizing antibody responses. Known VP5* antigenic epitopes [66] were mapped to the three-dimensional structure of VP4 from RRV, showing that the amino acid residue corresponding to E263 in VP4 from GR10924 is distant from these epitopes (Supplementary Figure S7). While the mutation identified in VP4 was outside of known VP4 antigenic epitopes, immunization and neutralization studies will have to be conducted to verify that this reassortant can induce cross-neutralizing antibodies. The triple-reassortant could also be used to further investigate factors that influence reassortment, e.g., compatibility with other human RVA VP7 genotypes or other human RVA structural and non-structural proteins. 

In conclusion, we have successfully generated a triple-reassortant of an African human RVA strain, where we included all major antigens into one virus by integrating the genome segments encoding human RVA P[6] VP4, G9 VP7 and I2 VP6 into the backbone of the simian RVA strain SA11. Additionally, we identified a mutation in human RVA P[6] VP4 that substantially improved replication in cell culture by a yet unknown mechanism, indicating that single-point mutations in human wildtype RVA VP4 genes can substantially improve cell culture replication. In the future, the use of this triple-reassortant as a potential next-generation vaccine strain should be investigated.

## Data Availability

All data are contained in the manuscript or are available upon reasonable request from the corresponding author.

## Supplementary Materials

The following supporting information can be downloaded at: https://www.mdpi.com/article/10.3390/v16040565/s1, Supplementary Information. Figure S1: Example of an RT-qPCR analysis of an SA11 NSP3-encoding plasmid standard for the determination of genome copy equivalents (GCEs)/ml culture supernatant. Figure S2: Repetition experiments for the generation of SA11 triple-reassortants containing VP4, VP7 and VP6 from three African human RVA strains. Figure S3: Sanger sequencing analyses of the VP4-encoding genome segment from the rSA11/triple-GR10924 reassortant that replicated to a higher titer in early passages. Figure S4: Repetition experiment for the rescue of SA11 mono- and triple-reassortants containing VP4 from human RVA strain GR10924 with and without the mutation A797G in VP4. Figure S5: Attempts to rescue of SA11 mono- and triple-reassortants containing VP4 from human RVA strains Moz60a or Moz308 with the mutation A797G in VP4. Figure S6: Mapping of E263, T42 and V322 to the VP4 trimeric spike. Figure S7: Mapping of known VP5* antigenic epitopes to the three-dimensional structure of VP4 from rhesus rotavirus (PDB 4v7q, chain BX). Table S1: Primer pairs used for RT-PCR analyses, Sanger sequencing and mutagenesis. Table S2: Average coverage, coverage range, and % ORF sequenced of rSA11/triple-GR10924 and rSA11/triple-GR10924-duplicate after next generation sequencing of virus present in the supernatant of the 10th passage. Table S3: UTRs of the genome segments encoding structural viral proteins from rSA11/triple-GR10924 and rSA11/triple-GR10924-duplicate. Table S4: UTRs of the genome segments encoding non-structural viral proteins from rSA11/triple-GR10924 and rSA11/triple-GR10924-duplicate.
