# Supplementary material for: VP4 Mutation Boosts Replication of Recombinant Human/Simian Rotavirus in Cell Culture
Source: Viruses. 2024 Apr 5;16(4):565. doi: 10.3390/v16040565 (PMC11054354; doi:10.3390/v16040565)
Supplement: Supplementary file 1 [file viruses-16-00565-s001.zip › viruses-2908892-supplementary.pdf]

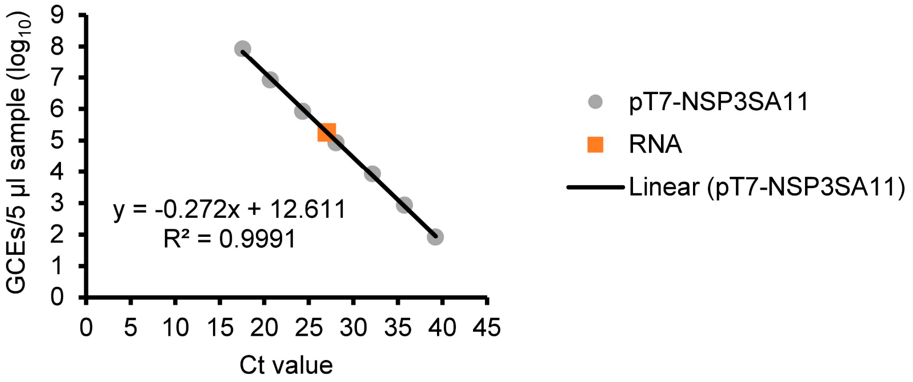

Figure S1. Example of an RT-qPCR analysis of an SA11 NSP3-encoding plasmid standard for the determination of genome copy equivalents (GCEs)/ml culture supernatant. A plasmid preparation of pT7-NSP3SA11 with a known number of GCEs (2.34×10^{11} copies/µg) was serially diluted and 5 µl were used in RT-qPCR analyses in parallel with 5 µl RNA isolated from culture supernatants of infected MA-104 cells. Linear regression analysis of the RT-qPCR results of the plasmid standard resulted in a formula enabling the calculation of GCEs contained in 5 µl RNA on the basis of the Ct values. The GCEs/ml culture supernatant were calculated by multiplying the number of GCEs contained in 5 µl RNA with factor A (to account for the volume of culture supernatant that RNA was prepped from), factor B (to account for the RNA elution buffer volume), and factor C (to account for the dilution due to DNase digest).

(a)

CPE	Mock	rSA11	rSA11/triple-GR10924	rSA11/triple-Moz60a	rSA11/triple-Moz308
P1	-	+	-	-	-
P2	-	NA	-	-	-
P3	-	NA	-	-	-

(b)

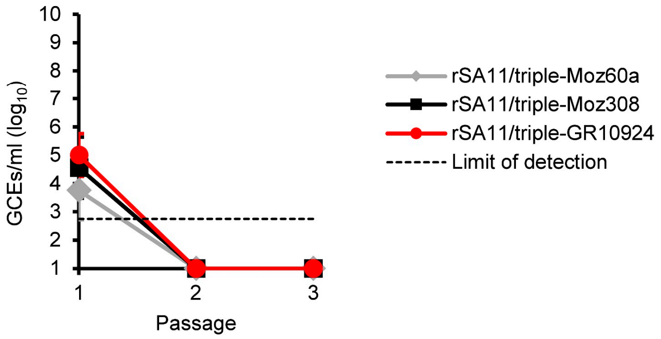

Figure S2. Repetition experiments for the generation of SA11 triple-reassortants containing VP4, VP7 and VP6 from three African human RVA strains. **(a)** Overview of the developed cytopathic effect (CPE) upon passaging in MA-104 cells. Data are representative of two independent experiments. **(b)** Analyses of the freeze-thaw supernatants by qRT-PCR after each passage in MA-104 cells. Data are means ± standard deviation from two independent experiments. Mock = Mock-infected cells; rSA11 = Recombinant SA11; rSA11/triple-GR10924, rSA11/triple-Moz60a and rSA11/triple-Moz308 = Recombinant rotaviruses carrying segment 4 (VP4), segment 9 (VP7) and segment 6 (VP6) from the indicated human RVA strain in the backbone of SA11; P1-3 = Passages 1-3; red minus = No CPE; green plus = Strong CPE; NA = Not analyzed; GCEs = Genome copy equivalents.

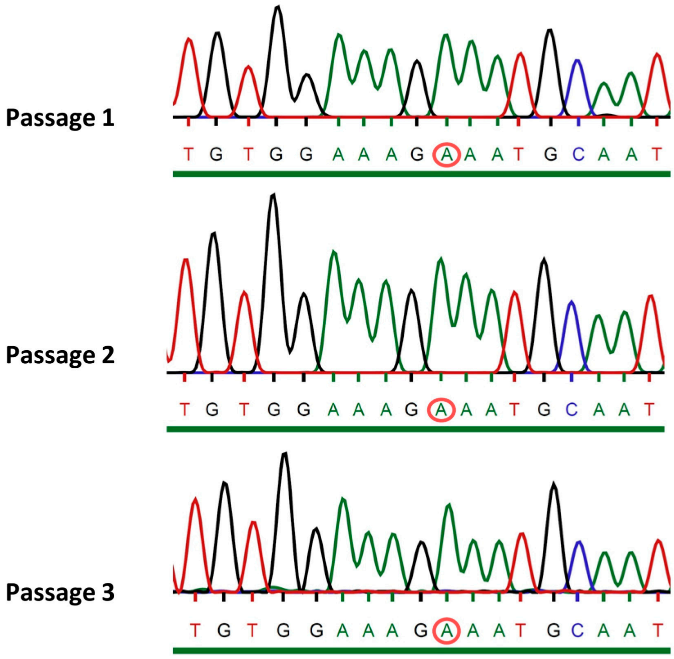

Figure S3. Sanger sequencing analyses of the VP4-encoding genome segment from the rSA11/triple-GR10924 reassortant that replicated to a higher titer in early passages. Sequencing chromatograms of passages 1-3 are shown. The read circle marks nucleotide position 797 in the VP4-encoding genome segment from human RVA strain GR10924. The green line below the chromatograms indicates that the probability for a wrong base call was equal to or less than 1 in 1000.

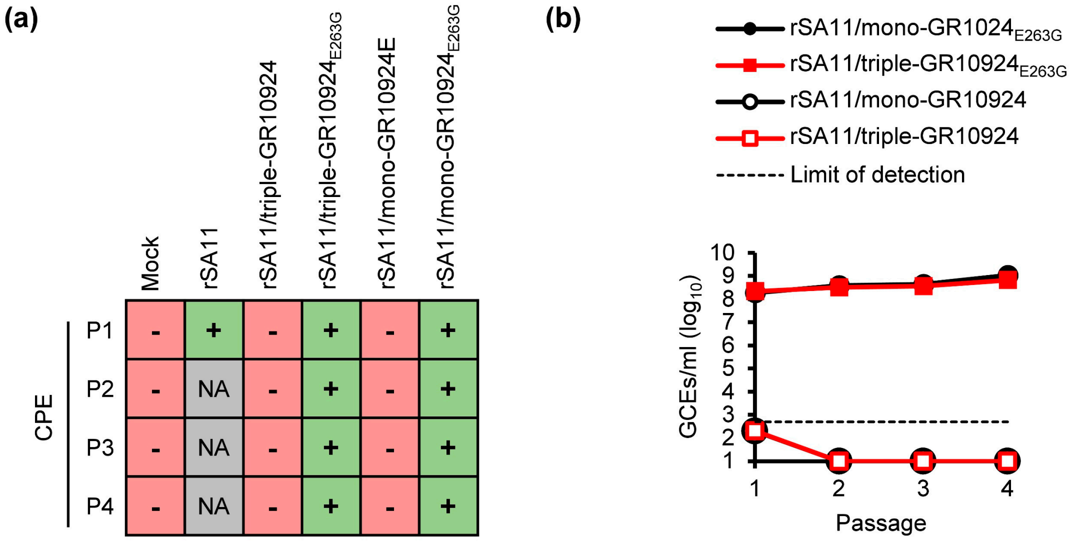

Figure S4. Repetition experiment for the rescue of SA11 mono- and triple-reassortants containing VP4 from human RVA strain GR10924 with and without the mutation A797G in VP4. **(a)** Overview of the developed cytopathic effect (CPE) upon passaging in MA-104 cells. **(b)** Analyses of the freeze-thaw supernatants by qRT-PCR after each passage in MA-104 cells. Mock = Mock-infected cells; rSA11 = Recombinant SA11; rSA11/triple-GR10924 = Recombinant rotavirus carrying segment 4 (VP4), segment 9 (VP7) and segment 6 (VP6) from human RVA strain GR10924 in the backbone of SA11; rSA11/triple-GR10924$_{E263G}$ = rSA11/triple-GR10924 with VP4-E263G; rSA11/mono-GR10924 = Recombinant rotavirus carrying segment 4 (VP4) from human RVA strain GR10924 in the backbone of SA11; rSA11/mono-GR10924$_{E263G}$ = rSA11/mono-GR10924 with VP4-E263G; P1-4 = Passages 1-4; red minus = No CPE; green plus = Strong CPE; NA = Not analyzed; GCE = Genome copy equivalents.

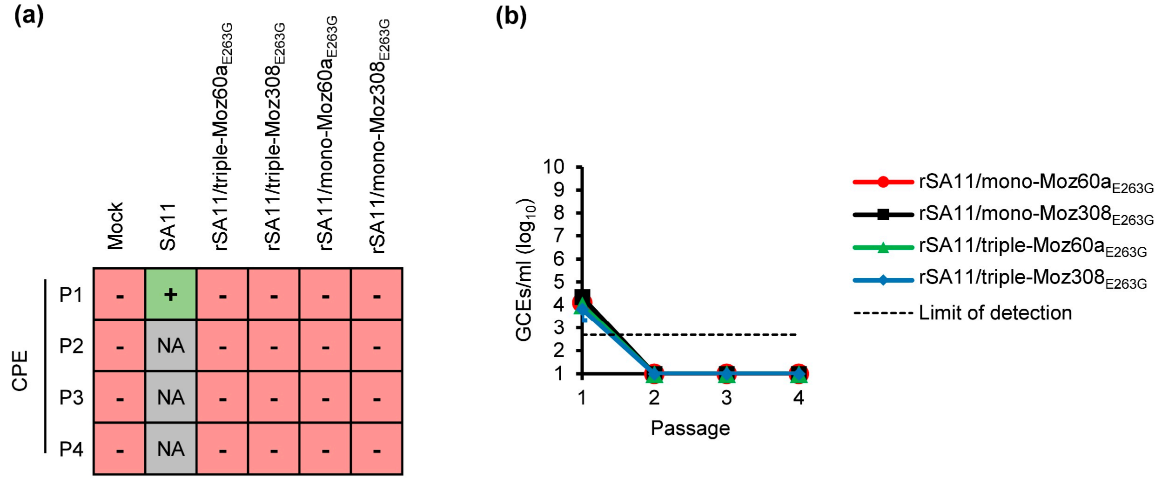

Figure S5. Attempts to rescue of SA11 mono- and triple-reassortants containing VP4 from human RVA strains Moz60a or Moz308 with the mutation A797G in VP4. **(a)** Cytopathic effect (CPE) upon passaging in MA-104 cells. The result is representative of one experiment performed in duplicates. **(b)** Determined number of GCEs/ml in freeze-thaw supernatants after each passage. Data are means ± standard deviation from one experiment performed in duplicates. rSA11/triple-Moz308$_{E263G}$, rSA11/triple-Moz60a$_{E263G}$ = Recombinant rotaviruses carrying segment 4 (VP4) with VP4-E263G, segment 9 (VP7) and segment 6 (VP6) from the indicated human RVA strain in the backbone of SA11; rSA11/mono-Moz308$_{E263G}$, rSA11/mono-Moz60a$_{E263G}$ = Recombinant rotaviruses carrying segment 4 (VP4) with VP4-E263G from the indicated human RVA strain in the backbone of SA11; P1-4 = Passages 1-4; red minus = No CPE; green plus = Strong CPE; NA = Not analyzed.

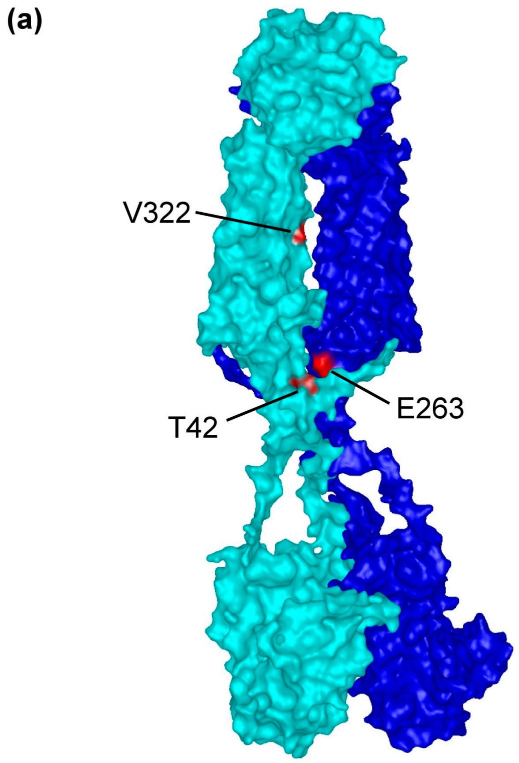

Figure S6. Mapping of E263, T42 and V322 to the VP4 trimeric spike. The three-dimensional structure of the VP4 spike on the basis of the atomic model of an infectious rhesus rotavirus particle (PDB 4v7q, chains BX, BY and BZ) is shown. VP4A, VP4B and VP4C are colored in blue, cyan and yellow, respectively. **(a)** The position of the amino acid residue corresponding to E263, T42 and V322 in VP4A and VP4B from GR10924 are highlighted in red. **(b)** The position of the amino acid residue corresponding to E263 and V322 in VP4C from GR10924 are highlighted in red.

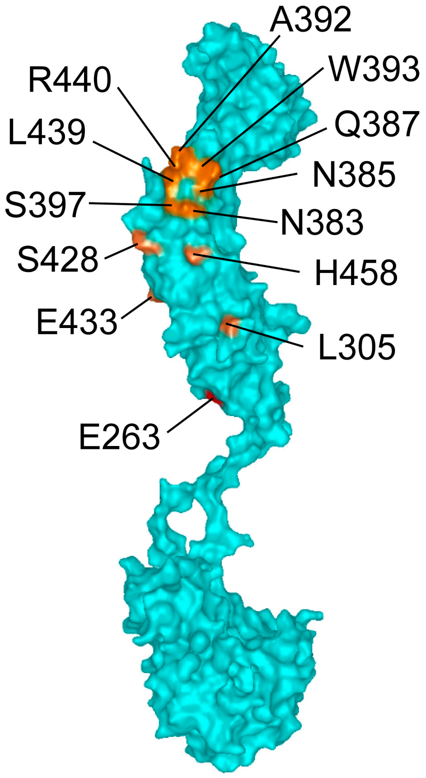

Figure S7. Mapping of known VP5* antigenic epitopes to the three-dimensional structure of VP4 from rhesus rotavirus (PDB 4v7q, chain BX). Antigenic epitopes are highlighted in orange and the amino acid residue as well as the position corresponding to VP4 from GR10924 are shown. The amino acid residue corresponding to E263 in VP4 from GR10924 is highlighted in red.

Table S1. Primer pairs used for RT-PCR analyses, Sanger sequencing and mutagenesis.

Primer	Sequence	Figure
VP4 GR10924-seq-f	5′-TGGGTTACCACCAATGCAGAA-3′	2b
VP4 GR10924-seq-r	5′-CACACCATTGACTGAGCAAGT-3′	2b
VP2 SA11-f	5′-CGTAATAGATGGTATTGGAAATT-3′	3c
VP2 SA11-r	5′-CTGGTATGTAATTGAAAATGATA-3′	3c
VP4 SA11-f	5′-ACGACAGTTGGACCATTGCT-3′	3c
VP4 SA11-r	5′-AGCGTTAGGTGTCTGTCCTTC-3′	3c
VP7 SA11-f	5′-TATTATCCGACTGAGGCTGCG-3′	3c
VP7 SA11-r	5′-GCAACGTCGCGTCATATTTCA-3′	3c
VP4 GR10924-f	5′-GATGAGTGGAGGTGCAGTGT-3′	3c
VP4 GR10924-r	5′-GCTGGTAGCCCGTACAATCC-3′	3c
VP7 GR10924-f	5′-ACCGATGTTGTTGATGGTGTG-3′	3c
VP7 GR10924-r	5′-ACATCTGAGCCACCGACTTG-3′	3c
VP6-seq-f	5′-CAAATGATAATTACTATGAATGGAAATGA-3′	3d
VP6-seq-r	5′-TAGAAACTCCACTTCAACGTTATT-3′	3d
VP4mut GR10924-f	5′-TGTGGAAAGGAATGCAATATAACAGAG-3′	n.s.
VP4mut GR10924-r	5′-ACGATGTCTTTGATATAATGATATCCTCAT-3′	n.s.
VP4mut Moz60a-f	5′-TATGGAAAGGAATGCAATATAACAGAGAT-3′	n.s.
VP4mut Moz60a-r	5′-ATGAAGTCTTTGAAATTGTAATGTCTTCATT-3′	n.s.
VP4mut Moz308-f	5′-TATGGAAGGGAATGCAATATAATAGAGATATT-3′	n.s.
VP4mut Moz308-r	5′-ATGAGGTTTTTGAAATTGTAATATCTTCATTAACT-3′	n.s.
VP4 Moz60a-seq-f	5′-TCCAAGGTCCCAAGAGTCTAA-3′	n.s.
VP4 Moz60a-seq-r	5′-GGTGGGTAAAGATCCTCCGT-3′	n.s.
VP4 Moz308-seq-f	5′-ACCACTGATAGATCAGATACTGC-3′	n.s.
VP4 Moz308-seq-r	5′-GTAATTTGTTCACCATCACGCA-3′	n.s.

n.s. – not shown

Table S2. Average coverage, coverage range, and % ORF sequenced of rSA11/triple-GR10924 and rSA11/triple-GR10924 duplicate after next generation sequencing of virus present in the supernatant of the 10th passage.

Segment	rSA11/triple-GR10924			rSA11/triple-GR10924 duplicate		
	Average coverage	Coverage range	% ORF sequenced	Average coverage	Coverage range	% ORF sequenced
NSP1	349	110-495	100.0	807	192-1169	100.0
NSP2	281	161-390	100.0	603	242-865	100.0
NSP3	299	97-397	100.0	537	154-713	100.0
NSP4	177	63-250	100.0	247	85-380	100.0
NSP5	122	46-157	100.0	222	54-278	100.0
VP1	233	20-325	100.0	528	109-723	100.0
VP2	224	34-361	100.0	508	87-727	100.0
VP3	234	69-363	100.0	528	201-763	100.0
VP4	205	4-345	100.0	499	2-664	99.9[A]
VP6	292	68-464	100.0	573	83-882	100.0
VP7	318	135-468	100.0	555	165-758	100.0

[A]First two nucleotides of the VP4 ORF missing

Table S3. UTRs of the genome segments encoding structural viral proteins from rSA11/triple-GR10924 and rSA11/triple-GR10924 duplicate. Accession numbers of reference sequences are shown in brackets. % UTR = percentage of the UTR identified by sequencing in relation to the reference sequence; NA = Not applicable.

Segment	Sequence 5‘ -> 3‘	% UTR
VP1_5'UTR (LC178564)	GGCTATTAAAGCTGTACA	NA
rSA11/triple-GR10924	AAGCTGTACA	55.6
rSA11/triple-GR10924 duplicate	AAGCTGTACA	55.6
VP1_3'UTR (LC178564)	AACGCTTAGATGTGACC	NA
rSA11/triple-GR10924	AACGCTTAGATGTGA	88.2
rSA11/triple-GR10924 duplicate	AACGCTTAGATGTGAC	94.1
VP2_5'UTR (LC178565)	GGCTATTAAAGGCTCA	NA
rSA11/triple-GR10924	GGCTCA	37.5
rSA11/triple-GR10924 duplicate	GGCTATTAAAGGCTCA	100.0
VP2_3'UTR (LC178565)	ACGCCAACCCCATTGTGGAGATATGACC	NA
rSA11/triple-GR10924	ACGCCAACCCCATTGTGGAGATA	82.1
rSA11/triple-GR10924 duplicate	ACGCCAACCCCATTGTGGAGATATG	89.3
VP3_5'UTR (LC178566)	GGCTATTAAAGCAGTACCAGTAGTGTGTTTTACCTCTGATGGTGTAAAC	NA
rSA11/triple-GR10924	AAGCAGTACCAGTAGTGTGTTTTACCTCTGATGGTGTAAAC	83.7
rSA11/triple-GR10924 duplicate	AGCAGTACCAGTAGTGTGTTTTACCTCTGATGGTGTAAAC	81.6
VP3_3'UTR (LC178566)	GCTAAAAACTTAACACACTAGTCATGATGTGACC	NA
rSA11/triple-GR10924	GCTAAAAACTTAACACACTAGTCATGATGTG	91.2
rSA11/triple-GR10924 duplicate	GCTAAAAACTTAACACACTAGTCATGATGTGACC	100.0
VP4_5'UTR (FJ183356)	GGCTATAAA	NA
rSA11/triple-GR10924	AAA	33.3
rSA11/triple-GR10924 duplicate	-	0.0
VP4_3'UTR (FJ183356)	GAGCTCTATAGAGGATGTGACC	NA
rSA11/triple-GR10924	GAGCTCTATAGAGGATGTGAC	95.5
rSA11/triple-GR10924 duplicate	GAGCTCTATAGAGGATGTGACC	100.0
VP6_5'UTR (FJ183358)	GGCTTTAAAACGAAGTCTTCAAC	NA
rSA11/triple-GR10924	CGAAGTCTTCAAC	56.5
rSA11/triple-GR10924 duplicate	GGCTTTAAAACGAAGTCTTCAAC	100.0
VP6_3'UTR (FJ183358)	GGACCAAGCTAACCACTTGGTATCCAACTTTGATGAGTATGTAGCTACGTC<br>AAGCTGTTTGAACTCTGTAAGTAAGGATGCGCTTACGTATTCGCTACACAG<br>AGTAATCACTCAGATGACGTAGTGAGAGGATGTGACC	NA
rSA11/triple-GR10924	GGACCAAGCTAACCACTTGGTATCCAACTTTGATGAGTATGTAGCTACGTC<br>AAGCTGTTTGAACTCTGTAAGTAAGGATGCGCTTACGTATTCGCTACACAG<br>AGTAATCACTCAGATGACGTAGTGAGAGGATGTGAC	99.3
rSA11/triple-GR10924 duplicate	GGACCAAGCTAACCACTTGGTATCCAACTTTGATGAGTATGTAGCTACGTC<br>AAGCTGTTTGAACTCTGTAAGTAAGGATGCGCTTACGTATTCGCTACACAG<br>AGTAATCACTCAGATGACGTAGTGAGAGGATGTGACC	100.0
VP7_5'UTR (FJ183360)	GGCTTTAAAAGAGAGAATTTCCGTTTGGCTAGCGGTTAGCTCCTTTTA	NA
rSA11/triple-GR10924	AGAGAATTTCCGTTTGGCTAGCGGTTAGCTCCTTTTA	77.1
rSA11/triple-GR10924 duplicate	AGAGAATTTCCGTTTGGCTAGCGGTTAGCTCCTTTTA	77.1
VP7_3'UTR (FJ183360)	TATATCTTAGGTTAGAATTGGTCGATGTGACC	NA
rSA11/triple-GR10924	TATATCTTAGGTTAGAATTGGTC	71.9
rSA11/triple-GR10924 duplicate	TATATCTTAGGTTAGAATTGGTCGATGTGA	93.8

Table S4. UTRs of the genome segments encoding non-structural viral proteins from rSA11/triple-GR10924 and rSA11/triple-GR10924 duplicate. Accession numbers of reference sequences are shown in brackets. % UTR = percentage of the UTR identified by sequencing in relation to the reference sequence; NA = Not applicable. The exchanged nucleotide in NSP4 is underlined and in bold.

Segment	Sequence 5‘ -> 3‘	% UTR
NSP1_5'UTR (LC178570)	GGCTTTTTTTTGAAAAGTCTTGTGTTAGCC	NA
rSA11/triple-GR10924	GAAAAGTCTTGTGTTAGCC	63,3
rSA11/triple-GR10924 duplicate	TTTTTTTTGAAAAGTCTTGTGTTAGCC	90,0
NSP1_3'UTR (LC178570)	AATTATGTCACTATCTAATTATACAGTATTTAGCCATCACAAGACCGTCCAG<br>ACTAGAGTAGCGCCTAGCTGGCAAAATACTGTGAACC	NA
rSA11/triple-GR10924	AATTATGTCACTATCTAATTATACAGTATTTAGCCATCACAAGACCGTCCAG<br>ACTAGAGTAGCGCCTAGCTGGCAAAATACTGTG	95.5
rSA11/triple-GR10924 duplicate	AATTATGTCACTATCTAATTATACAGTATTTAGCCATCACAAGACCGTCCAG<br>ACTAGAGTAGCGCCTAGCTGGCAAAATACTGTGAAC	98.9
NSP2_5'UTR (LC178571)	GGCTTTTAAAGCGTCTCAGTCGCCGTTTGAGCCTTGCGGTGTAGCC	NA
rSA11/triple-GR10924	AGCGTCTCAGTCGCCGTTTGAGCCTTGCGGTGTAGCC	80,4
rSA11/triple-GR10924 duplicate	CGTCTCAGTCGCCGTTTGAGCCTTGCGGTGTAGCC	76,1
NSP2_3‘UTR (LC178571)	TTCGCTATCAATTTGAGGATGATGATGGCTTAGCAAGAATAGAAAGCGCTTA<br>TGTGACC	NA
rSA11/triple-GR10924	TTCGCTATCAATTTGAGGATGATGATGGCTTAGCAAGAATAGAAAGCGCTTA<br>TGTGACC	100.0
rSA11/triple-GR10924 duplicate	TTCGCTATCAATTTGAGGATGATGATGGCTTAGCAAGAATAGAAAGCGCTTA<br>TGTGACC	100.0
NSP3_5'UTR (LC178572)	GGCATTTAATGCTTTTCAGTGGTTG	NA
rSA11/triple-GR10924	AATGCTTTTCAGTGGTTG	72.0
rSA11/triple-GR10924 duplicate	GGCATTTAATGCTTTTCAGTGGTTG	100.0
NSP3_3'UTR (LC178572)	CCATTTTGATACATGTTGAACAATCAAATACAGTGTTAGTATGTTGTCATCT<br>ATGCATAACCCTCTATGAGCACAATAGTTAAAAGCTAACACTGTCAAAAACC<br>TAAATGGCTATAGGGGCGTTATGTGGCC	NA
rSA11/triple-GR10924	CCATTTTGATACATGTTGAACAATCAAATACAGTGTTAGTATGTTGTCATCT<br>ATGCATAACCCTCTATGAGCACAATAGTTAAAAGCTAACACTGTCAAAAACC<br>TAAATGGCTATAGGGGCGTTATGT	97.0
rSA11/triple-GR10924 duplicate	CCATTTTGATACATGTTGAACAATCAAATACAGTGTTAGTATGTTGTCATCT<br>ATGCATAACCCTCTATGAGCACAATAGTTAAAAGCTAACACTGTCAAAAACC<br>TAAATGGCTATAGGGGCGTTATG	96.2
NSP4_5'UTR (LC178573)	GGCTTTTAAAAGTTCTGTTCCGAGAGAGCGCGTGCGGAAAG	NA
rSA11/triple-GR10924	CTGTTCCGAGAGAGCGCGTGCGGAAAG	65.9
rSA11/triple-GR10924 duplicate	AGTTCTGTTCCGAGAGAGCGCGTGCGGAAAG	75.6
NSP4_3'UTR (LC178573)	GAGGTTGAGCTGCCGTCGACTGTCCTCGGAAGCGGCGGAGTTCTTTACAGTA<br>AGCACCATCGGACCTGATGGCTGACTGAGAAGCCACAGTCAGCCATATCGCG<br>TGTGGCTCAAGCCTTAATCCCGTTTAACCAATCCGGTCAGCACCGGACGTTA<br>ATGGAAGGAACGGTCTTAATGTGACC	NA
rSA11/triple-GR10924	GAGGTTGAGCTGCCGTCGACTGTCCTCGGAAGCGGCGGAGTTCTTTACAGTA<br>AGCACCATCGGACCTGATGGCTGACTGAGAAGCCACAGTCAGCCATATCGCG<br>TGTGGCTCAAGCCTTAATCCCGTTTAACCAATCCGGTCAGCACCGGACGTTA<br>ATGGAAGGAACGG	92.9
rSA11/triple-GR10924 duplicate	GAGGTTGAGCTGCCGTCGACTGTCCTCGGAAGCGGCGGAGTTCTTTACAGTA<br>AGCACCATCGGACCTGATGGCTGACTGAGAAGCCACAGTCAGCCATATCGCG<br>TGTGGCTCAAGCCTTAATCCCGTTTAACCAA**c**CGGTCAGCACCGGACGTTAA<br>TGGAAGGAAC	91.2
NSP5_5'UTR (LC178574)	GGCTTTTAAAGCGCTACAGTG	NA
rSA11/triple-GR10924	GCGCTACAGTG	56.9
rSA11/triple-GR10924 duplicate	GCGCTACAGTG	75.6
NSP5_3'UTR (LC178574)	GTCTGACCTGGGAACACACTAGGGAGCTCCCCACTCCCGTTTTGTGACC	NA
rSA11/triple-GR10924	GTCTGACCTGGGAACACACTAGGGAGCTCCCCACTCCCGTT	83.7
rSA11/triple-GR10924 duplicate	GTCTGACCTGGGAACACACTAGGGAGCTCCCCACTC	73.5
